# Topology Optimization Method of a Cavity Receiver and Built-In Net-Based Flow Channels for a Solar Parabolic Dish Collector

**DOI:** 10.3390/e25030398

**Published:** 2023-02-22

**Authors:** Jun Liu, Renfu Li, Yuxuan Chen, Jianguo Zheng, Kun Wang

**Affiliations:** 1 School of Aerospace Engineering, Huazhong University of Science and Technology, Wuhan 430074, China; 2 Wuhan Secondary Ship Design & Research Institute, Wuhan 430064, China; 3 School of Energy and Power Engineering, Huazhong University of Science and Technology, Wuhan 430074, China

**Keywords:** parabolic dish collector, thermal–fluid, topology optimization, multi-objective optimization, genetic algorithm

## Abstract

The design of a thermal cavity receiver and the arrangement of the fluid flow layout within it are critical in the construction of solar parabolic dish collectors, involving the prediction of the thermal–fluid physical field of the receiver and optimization design. However, the thermal–fluid analysis coupled with a heat loss model of the receiver is a non-linear and computationally intensive solving process that incurs high computational costs in the optimization procedure. To address this, we implement a net-based thermal–fluid model that incorporates heat loss analysis to describe the receiver’s flow and heat transfer processes, reducing computational costs. The physical field results of the net-based thermal–fluid model are compared with those of the numerical simulation, enabling us to verify the accuracy of the established thermal–fluid model. Additionally, based on the developed thermal–fluid model, a topology optimization method that employs a genetic algorithm (GA) is developed to design the cavity receiver and its built-in net-based flow channels. Using the established optimization method, single-objective and multi-objective optimization experiments are conducted under inhomogeneous heat flux conditions, with objectives including maximizing temperature uniformity and thermal efficiency, as well as minimizing the pressure drop. The results reveal varying topological characteristics for different optimization objectives. In comparison with the reference design (spiral channel) under the same conditions, the multi-objective optimization results exhibit superior comprehensive performance.

## 1. Introduction

Concentrated solar power (CSP) is an important technology for realizing solar thermal power generation [1,2,3] and solar thermal energy storage [4,5]. The parabolic or hyperboloid reflecting dish is used to focus the solar radiation, and the thermal cavity receiver is used to absorb heat to provide a high-temperature working medium, which can be used as the thermal energy storage component [6]. The parabolic dish collector (PDC) is one of the main types of CSP systems. However, the concentrated solar flux distribution in the CSP system is extremely non-uniform, which causes local high temperatures and large temperature gradients in the receiver and seriously affects the safety performance and service life of the CSP system [7,8,9]. The typical problems include degeneration of the materials, thermal stress, deformation, and overburning [10].

At present, parametric research on improving the thermal performance of the receiver mainly focuses on the influence of the receiver’s shape, geometric parameters of the receiver, and tube diameter on its thermal performance. In the early stages, most studies focused on the influence of the cavity shape of the receiver, such as cylindrical, cuboidal, hemispherical, conical, and other irregular shapes [11,12,13]. For instance, Karwa et al. [14] studied receiver shape optimization and proposed a new receiver design for a compound parabolic concentrator. For a given cavity shape, many scholars [15,16,17,18] have studied the influence of various geometric parameters such as cavity diameter and cavity height on the thermal performance of the receiver. Additionally, the heat transfer ability of the heat transfer fluid (HTF) in heat transfer tubes should match the solar flux distribution as closely as possible. Hence, it is important to improve the structure and layout of the heat transfer tube to improve these problems caused by nonuniform heat flux distribution. Regarding tube structure, some have studied the influence of tube diameter [17,19,20], and some scholars have investigated the influence of tube layout, such as the tube loop number [15]. For example, Wang et al. [21] employed an asymmetric outward convex corrugated tube as the metal tube of a parabolic trough receiver to enhance the tube receiver’s overall heat transfer efficacy and dependability.

The above studies were quantitative studies on the geometric parameters of the receiver and tubes. In addition, some scholars have carried out optimization research on the thermal performance of the receiver. Since uniform temperature distribution in solar dish receivers and high optical–thermal efficiency are crucial to improving the reliability and economy of a solar dish system, Li et al. [22] designed a new solar receiver–Stirling heater configuration to obtain a uniform distribution over the tube walls. Moreover, phase change materials were employed to improve thermal uniformity. For example, a coupling heat transfer containing phase change was applied in a cavity receiver by Tao et al. [23], and could reduce the temperature gradient; in particular, it enhanced the thermal conductivity of the phase change material. In addition, heat pipe technology [24] has also been applied to achieve more uniformity in the temperature of receivers. The impact of metal foam inserts in the receiver tube of the parabolic trough collector was investigated by Wang et al. [25] regarding heat transfer under non-uniform heat flux boundary conditions. Moreover, some studies have adopted optimization algorithms to improve the performance of the receiver. Zheng et al. [26,27] found that the majority of optimization investigations on the porous configuration of heat transfer tubes have relied on a “parameter analysis” trial-and-error approach. In response to this, they put forward a new optimization methodology that combines computational fluid dynamics (CFD) and a genetic algorithm (GA) to optimize the receiver’s porous configurations. Shen et al. [28] developed a gradually varied porous configuration to boost the thermal performance of the porous volumetric solar receiver. They employed an optimization technique involving GA and CFD to establish the optimal distribution of porosity. Guo et al. [29] employed multi-parameter optimization of a parabolic trough solar receiver based on a GA. Du et al. [30] proposed an optimization method that couples the GA and the heat transfer analysis of the porous volumetric solar receiver. In their work, the receiver with relatively lower flow resistance and relatively higher thermal efficiency was obtained using multi-objective optimization (MOO). Risi et al. [31] considered solar thermal efficiency as an objective function with four design variables, and a GA was used for the optimization process. Moloodpoor et al. [32] developed an effective approach to solving the governing equations of heat transfer in parabolic trough collectors and used integrated particle swarm optimization to optimize the system’s thermal characteristics. Zadeh et al. [33] used the hybrid optimization algorithm including a GA and an SQP (sequence quadratic program), to improve the thermal performance of the solar parabolic trough collector, where the tube diameter, HTF velocity, etc., were set as design variables.

In summary, most current research is generally limited to optimizing the geometric parameters of the receiver and those of the tubes in it. However, a reasonable layout design of the fluid flow in the thermal cavity receiver is also crucial, which can be classified as a topology optimization problem. In recent years, there have been few studies on topology optimization of the heat transfer tube in a cavity receiver. Montes et al. [34] optimized the fluid flow pattern of a solar central receiver by adjusting the width and diameter of each pass, as well as the number of tubes to achieve a more uniform temperature distribution at the outlets of all circuits. However, Montes et al.’s design procedure did not adopt an optimization algorithm. In this article, we consider developing a topology optimization method to optimize the topology of flow layout while optimizing the geometric parameters of the receiver, to further improve the thermal and flow performance of the receivers.

In this study, fluid flow, heat transfer, and heat loss analysis are considered together to describe the heat transfer process in the receiver. However, the mathematical model of heat loss analysis based on previous studies [32,35,36] is highly nonlinear, and the conventional thermal–fluid coupling model based on the finite element method (or finite volume method) is also very computationally expensive. The optimization of thermal–fluid analysis coupled with the heat loss model in a receiver will be a nonlinear and computationally intensive process. The “ground structure method” was proposed by Dorn et al. [37]. In this method, the initial design domain is discretized into enough units, and then some units are removed or added using optimization algorithms to realize topology optimization. Previous work [38] adopted the network structure as the ground structure for thermal–fluid analysis and optimization, to reduce the computational cost. The network structure comprises a series of nodes and edges that connect those nodes. The original complex flow calculation can be simplified into one-dimensional flow, and the temperature field is calculated based on the finite difference method, which can greatly reduce the requirement for computing resources. In addition, many studies have taken the thermal performance of receiver as the optimization objective, while few optimizations have adopted flow dissipation as the objective. In this work, the MOO is performed, considering both thermal performance and flow energy dissipation under nonuniform heat flux, and a Pareto front is obtained. A GA is utilized as the optimizer tool, as it is an efficient metaheuristic optimization method to solve MOO problems [39].

The remainder of this paper is organized as follows. In Section 2, the net-based thermal–fluid model and heat loss model are introduced. In Section 3, the objective function, design variables, and implementation of optimization by the GA are provided. In Section 4.1, the relationship between heat loss and cavity temperature, and the relationship between thermal efficiency and receiver size, are discussed. In Section 4.2, a comparative numerical example under uniform heat flux is carried out to verify the accuracy of the model in Section 2. In Section 4.3, the optimization results of the GA under inhomogeneous heat flux are obtained and compared with the helical channel as a reference design. Finally, the conclusions are presented in Section 5.

## 2. Physical Model

### 2.1. Net-Based Thermal–Fluid Model

**Figure 1:**
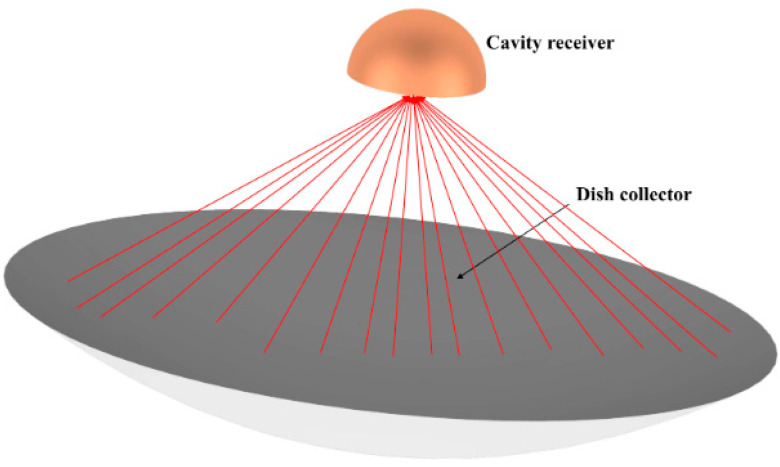
Three-dimensional schematic for the hemispherical-shaped receiver and the dish collector under it.

A hemispherical design shown in Figure 1 is adopted as the receiver shape in this paper. However, the physical model is not limited by the shape of the receiver. The purpose of the conventional arrangement of receiver tubes in the current research is to directly use the tube wall as the heated surface to receive solar radiation. However, this will lead to a large temperature gradient in the tube wall, resulting in considerable thermal stress. In some studies [35], the heating surface is arranged on the underside of the tube, which transfers heat to the tube wall after being heated, and then heats the fluid. This may lead to insufficient contact between the tube wall and the heating surface due to the thermal contact resistance between the two surfaces. In this paper, a new thermal receiver with built-in channels is proposed, in which the fluid flows directly into the channel grooves in the solid to exchange heat between the fluid and solid; this can reduce the temperature difference between the inside and the outside. The embedded complex channel structure can be easily fabricated using additive manufacturing technology. It is further optimized to obtain the maximum thermal efficiency and thermal uniformity based on this channel structure. The embedded heat transfer tube is hereinafter referred to as the heat transfer channel to distinguish it from the tube structure.

**Figure 2:**
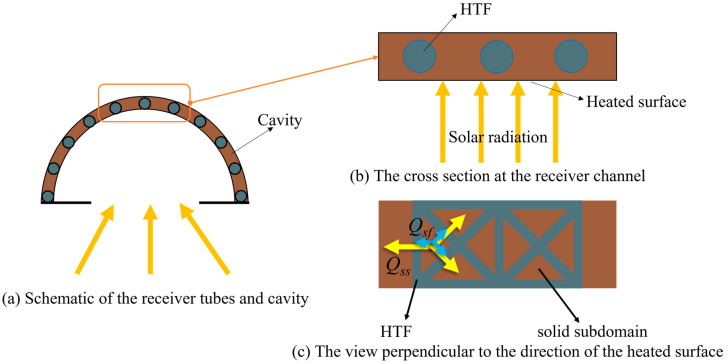
Network-type flow channel and heat transfer model, where $Q_{ss}$ represents the heat exchange between solid subdomains and *Q_sf_* represents the heat exchange between solid subdomains and HTF.

As shown in Figure 2, the entire design domain consists of solid subdomains divided by network-type channels filled with HTF. The one-dimensional pipe-net system is adopted in the fluid domain attached to the solid domain and the latter is divided into discrete elements. In the net-based thermal–fluid model [38], the network-type channel flow system with an incompressible steady fluid flow can be approximated as a pipe-net system and the channel wall is smooth. The governing equations for the pipe-net system are as follows [40]:

(1) $$\sum_{j=1}^{m}q_{j}=0$$

(2) $$\Delta p=rq^{2}$$

(3) $$\sum \Delta p_{i}=0$$

where q, Δp, and r represent the volume flow, friction loss (pressure drop), and friction factor of each channel, respectively. m is the number of nodes formed by each flow branch.

It can be seen from Equation (2) that the control equation for fluid flow is quadratic, which is difficult to solve directly. Therefore, the quasi-linear iterative method [38,40] is adopted to solve the nodal pressure, and then the flow distribution is obtained to simplify the calculation and increase the solution speed. The flow equation can be rewritten in matrix form as:

(4) Kp=0

where **p** is the pressure vector of each node and **K** is the friction factor matrix.

In the heat transfer model shown in Figure 2, the heat exchange is assumed to be in steady state. The fluid temperature in each channel is linearly distributed and the temperature of each solid subdomain is approximately concentrated at the center of the subdomain. According to the energy conservation equation, the equations for the temperature can be obtained:

(5) AT=f(T)

where A is the coefficient matrix for the heat transfer model, f(T) is the load vector of the heat source, which can be defined as a function of the temperature variable, T is the temperature vector, and T refers to the temperature variable.

**Figure 3:**
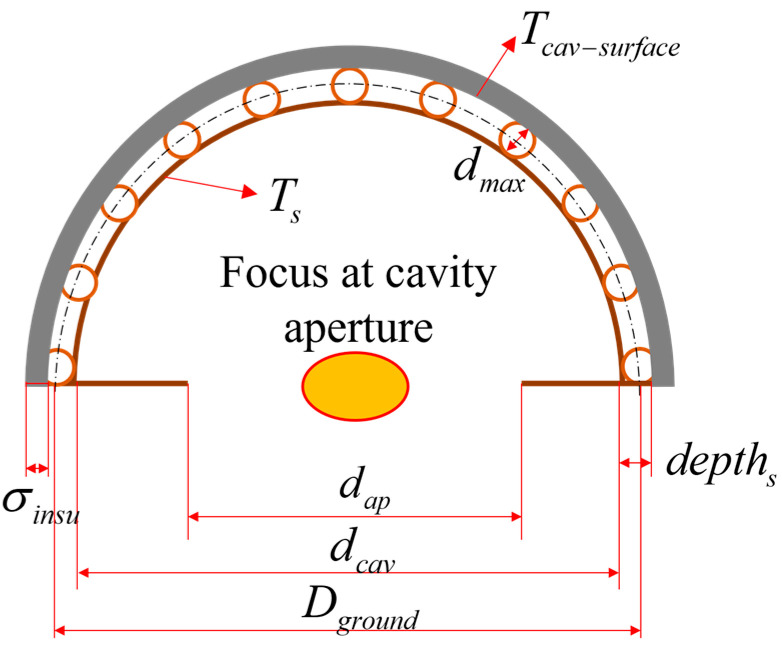
The relationship between the geometrical and temperature parameters of the receiver.

Moreover, the channels filled with HTF that are adopted in this paper are embedded in the solid domain of the cavity (see Figure 3), so the structure of the receiver cavity has the following geometric relationship:

(6) $$D_{ground}=d_{cav}+depth_{s}$$

(7) $$D_{out}=d_{cav}+2\times depth_{s}+2\times \sigma_{insu}$$

where $D_{ground}$ is the diameter of the ground structure for the net-based thermal–fluid model, that is, the diameter of the hemisphere formed by the center points of the cross-section of the channels. Moreover, $d_{cav}$ is the cavity diameter, $depth_{s}$ is the depth of the cavity, $D_{out}$ is the exterior surface diameter of the receiver cavity, and $\sigma_{insu}$ is the thickness of the insulation.

### 2.2. Heat Loss Model

The heat loss model should be considered to evaluate the thermal efficiency of the receiver, which consists of the following three parts: conduction heat loss, convection heat loss, and radiation heat loss. The equations of conduction heat loss are referenced from [35,41], while those of heat loss through convection and radiation are referenced from [35]. Convection and radiation losses generally contribute more to the total heat loss through the receiver than conduction loss [36,42]. However, they all need to be discussed separately because together they make up the heat loss of the receiver. Moreover, the temperature relationship shown in Equation (8) is assumed to facilitate the solution. The temperature distribution is shown in Figure 3, where *T_s_* is the interior cavity surface temperature, $T_{cav-surface}$ is the exterior cavity surface temperature, and $T_{s-ave}$ is the average cavity surface temperature. The temperature difference between the interior and exterior surfaces of the cavity is quite small since an embedded channel structure is adopted (see the verification example in Section 4.2). In the heat loss model, the temperature inside and outside the cavity is regarded as approximately equal, as shown in the following equation:

(8) $$T_{s}=T_{cav-surface}=T_{s-ave}$$

#### 2.2.1. Conduction Heat Loss

Conduction heat loss occurs in the insulation that wraps around the outside of the cavity, which can be calculated as follows:

(9) $$Q_{cond}=\frac{A_{s}\left(T_{s-ave}-T_{\infty }\right)}{\left(\frac{1}{h_{out}}+\frac{\delta_{insu}}{k_{insu}}\right)},$$

where *A*_*s*_ is the mean surface area, $T_{\infty }$ is the ambient temperature, $\sigma_{insu}$ is the thickness of the insulation, and $k_{insu}$ is the thermal conductivity of the insulation. $h_{out}$ is the heat transfer coefficient at the exterior of the insulated receiver, which can be obtained using the following:

(10) $$h_{out}=\frac{k_{\infty }Nu_{combined}}{D_{out}},$$

where $k_{\infty }$ is the thermal conductivity of atmospheric air. The combined Nusselt number can be calculated as:

(11) $$Nu_{combined}={\left(Nu_{forced}^{3.5}+Nu_{natural}^{3.5}\right)}^{\frac{1}{3.5}}.$$

The Nusselt numbers for forced and natural convection are calculated as:

(12) $$Nu_{forced}=2+\left[0.4Re^{0.5}+0.06Re^{0.67}\right]Pr^{0.4}{\left(\frac{\mu_{\infty }}{\mu_{s}}\right)}^{0.25},$$

(13) $$Nu_{natural}=2+\frac{0.589Ra^{0.25}}{{\left[1+{\left(\frac{0.469}{Pr}\right)}^{\frac{9}{16}}\right]}^{\frac{4}{9}}},$$

where Re is the Reynolds number, which can be obtained as follows:

(14) $$Re=\frac{\rho_{air}V_{wind}D_{r-out}}{\mu_{air}}.$$

For Equations (12)–(14), $\rho_{air}$, $\mu_{air}$, Pr, and Ra are the density, dynamic viscosity, Prandtl number, and Rayleigh number of air, respectively. $V_{wind}$ is the wind velocity. $\mu_{\infty }$ is the dynamic viscosity of wind at atmospheric temperature and $\mu_{s}$ is the dynamic viscosity of wind at surface temperature.

#### 2.2.2. Convection Heat Loss

Convection heat loss comes from natural convection and forced convection. Natural convection loss occurs in the air in the cavity, and is caused by the temperature difference between the cavity’s inner surface and the ambient air temperature. Forced convection occurs in the air outside the receiver because of wind speed. Furthermore, due to the difference between the temperature of the focal medium and the temperature of the wind, natural convection and forced convection occur when wind at ambient temperature blows through the inside and outside of the receiver aperture. The two convection losses can be expressed as:

(15) $$Q_{natural-conv}=h_{nat-conv}A_{cavity-surface}\left(T_{s}-T_{\infty }\right),$$

(16) $$Q_{forced-conv}=h_{forced-wind}A_{cavity-surface}\left(T_{cav-surface}-T_{\infty }\right).$$

The heat transfer coefficient for natural convection is determined by the following equation:

(17) $$Nu_{nat-conv}=0.534Gr^{0.218}{\left(1+\cos\theta \right)}^{0.916}{\left(1+\varepsilon \right)}^{0.473}{\left(\frac{N_{RC}}{N_{RC}+1}\right)}^{1.213}T_{R}{}^{0.082}{\left(\frac{d_{ap}}{d_{cav}}\right)}^{0.099},$$

(18) $$h_{nat-conv}=\frac{Nu_{nat-conv}k_{f}}{D},$$

where $\theta =45^{o}$ is the receiver inclination angle, set as a fixed value in this article, ε is the emissivity of receiver surface, $d_{ap}$ is the receiver aperture diameter, $T_{R}=\frac{T_{\infty }}{T_{s}}$ is the temperature ratio, $k_{f}$ is the thermal conductivity of air, $A_{cavity-surface}$ is the surface area of the receiver cavity, and $T_{focus}$ is the temperature of the medium at the focus. The Grashhof number is defined as follows:

(19) $$Gr=\frac{g\cdot \beta \cdot \left(T_{cav}-T_{amb}\right)\cdot d_{cav}^{3}}{\mu_{cav}^{2}},$$

where $T_{amb}$ is the ambient temperature, β is the volume expansion coefficient and $N_{RC}$ is the radiation conduction number, which can be calculated as:

(20) $$N_{RC}=\frac{\sigma \cdot T_{s}^{4}\left(\frac{D_{out}}{2}\right)}{\left(T_{s}-T_{\infty }\right)k_{\infty }}.$$

The forced convection heat transfer coefficient due to wind is determined as follows:

(21) $$h_{forced-wind}=2.8+3V_{wind},$$

where $V_{wind}$ is the velocity of the wind.

#### 2.2.3. Radiation Heat Loss

The radiation heat loss is the sum of emission heat loss and refection heat loss. Radiative loss due to emission by the receiver aperture is caused by the temperature difference between the cavity’s inner surface and the outside environment. Emission heat loss is radiated out through the receiver aperture. Reflection heat loss comes from the solar energy that is not absorbed but reflected after entering the cavity. It also enters the external environment through the receiver aperture.

Radiation heat loss due to emission is calculated as follows:

(22) $$Q_{rad-em}=A_{o}\varepsilon_{eff}F\sigma \left(T_{s}^{4}-T_{amb}^{4}\right),$$

where $A_{0}$ is the area of the receiver aperture, *F* is the view factor, σ=5.67e−8 W/(m^2^ K^4^) is the Stefan Boltzmann Constanta, and $\varepsilon_{eff}$ is the effective emissivity of the receiver surface, estimated using the following equation:

(23) $$\varepsilon_{eff}=\frac{\varepsilon_{s}}{1-\left(1-\varepsilon_{s}\right)(1-\left(\frac{d_{ap}}{d_{cav}}{)}^{2}\right)}.$$

where $\varepsilon_{s}$ is the emissivity of the cavity surface.

Radiative loss due to refection is estimated as follows:

(24) $$Q_{rad-ref}=\left(1-\alpha_{eff}\right)Q_{rad-b}.$$

The effective absorptivity $\alpha_{eff}$ and the radiative energy entering the receiver aperture $Q_{rad-b}$ are described as follows:

(25) $$\alpha_{eff}=\frac{\alpha_{s}}{1-\left(1-\alpha_{s}\right)(1-\left(\frac{d_{ap}}{d_{cav}}{)}^{2}\right)},$$

(26) $$Q_{rad-b}=Q_{b}A_{mirror}\tau_{i}\tau_{shad.}\rho_{mirror},$$

where $\alpha_{s}$ is the absorptivity of the radiative surface, $Q_{b}$ is the solar beam radiation energy, $A_{mirror}$ is the mirror surface, $\tau_{i}$ is the intercept factor, $\tau_{shad}$ is the shading factor, and $\rho_{mirror}$ is the mirror reflectivity.

#### 2.2.4. Thermal Efficiency

The thermal efficiency can be obtained from the following:

(27) $$\eta_{th}=\frac{Q_{use}}{Q_{rad-b}}=\frac{Q_{rad-b}-\left(Q_{cond}+Q_{conv}+Q_{rad}\right)}{Q_{rad-b}},$$

where $Q_{conv}=Q_{natural-conv}+Q_{forced-conv}$ and $Q_{rad}=Q_{rad-em}+Q_{rad.ref}$.

Consequently, the value of heat loss is highly nonlinear with the cavity temperature from the relationship of the heat loss model. This presents a challenge for coupling the heat loss model with the thermal–fluid model. In this paper, an appropriate fitting method is adopted to fit the original complex relationship into a linear relationship, details of which can be seen in Section 4.1.1.

## 3. Formulation of the Optimization Problem

This section describes the formulation of the optimization procedure. The optimization problem requires minimizing the objective values corresponding to temperature and the objective value fluid flow of the receiver under the given constraints.

### 3.1. Objective Function

Three cases are considered in the optimization problem: two single-objective optimizations with respect to temperature, and a multi-objective optimization (MOO) considering temperature and pressure drop.

$J_{1}$ is the first objective function, set as the standard deviation of the solid domain temperature, and is shown as follows:

(28) $$J_{1}=\sqrt{\frac{\sum_{i=1}^{N_{e}}{\left(T_{i}-\overset{-}{T}\right)}^{2}}{N_{e}-1}},$$

where $T_{i}$ is the temperature of each solid subdomain, $\overset{-}{T}$ is the average temperature of the whole solid region, and $N_{e}$ represents the number of solid subdomains.

$J_{2}$ is the second objective function used to reflect thermal efficiency, as shown in Equation (29). It is set to $1-\eta_{th}$ to match the format requirement of the objective function in the optimization algorithm.

(29) $$J_{2}=1-\eta_{th}=\frac{Q_{cond.}+Q_{conv.}+Q_{rad.}}{Q_{rad-b}}.$$

For the MOO, the objective is defined as:

(30) $$J=\left[J_{1}/Std_{0},\;J_{2}/\left(1-\eta_{0}\right),\;∆P/∆P_{0}\right],$$

where $Std_{0}$, $\eta_{0}$, and $∆P_{0}$ are the $J_{1}$ value, thermal efficiency, and total pressure drop of the initial structure, respectively. ∆P is the total pressure drop of the optimization result, indicating the flow consumption.

### 3.2. Design Variables

The geometric parameters of the receiver and built-in channels within it are determined by the design variables ξ used in the optimization problem. The geometric parameters include channel diameter (d), cavity diameter ($d_{cav}$), aperture diameter ($d_{ap}$), and thickness of insulation ($\delta_{insu}$). The lower and upper bounds of the geometric parameters used in these optimization examples are shown in Table 1, and are determined based on the analysis in Section 4.1.2. The determination of the upper and lower limits is based on the following considerations: In Section 4.1.1, the relationship between the temperature and thermal efficiency of the receiver is investigated using a cavity size of $\sigma_{insu}=0.05\;m$, $d_{ap}=0.18\;m$, and $d_{cav}=0.30\;m$ as an example. Although this relationship is not limited to this specific cavity size, the range of size changes is still centered around these geometric parameters in the subsequent study of the size–thermal efficiency relationship of the receiver in Section 4.1.2. For instance, the aperture diameter is kept constant at 0.18 m when examining the relationship between the cavity diameter and thermal efficiency. It should be noted that the dimensions are interdependent, and that, for example, the cavity diameter must be greater than the aperture diameter. Moreover, the upper and lower bounds of the design variables are taken from the ascending portion of the graph presented in Section 4.1.2, except for the variable of the aperture diameter.

### 3.3. Implementation of Optimization by GA

**Figure 4:**
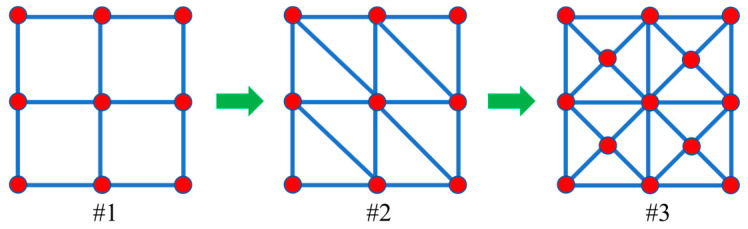
Schematic illustration of a net-based channel structure for the optimization problem.

The net-based channel structure #3, as shown in Figure 4, is selected to serve as a substructure of the ground structure in the subsequent optimization problem. Specifically, this structure will be employed to discretize the entire design domain. This structure can increase the possibility of channel orientation and increase the space for feasible solutions.

**Figure 5:**
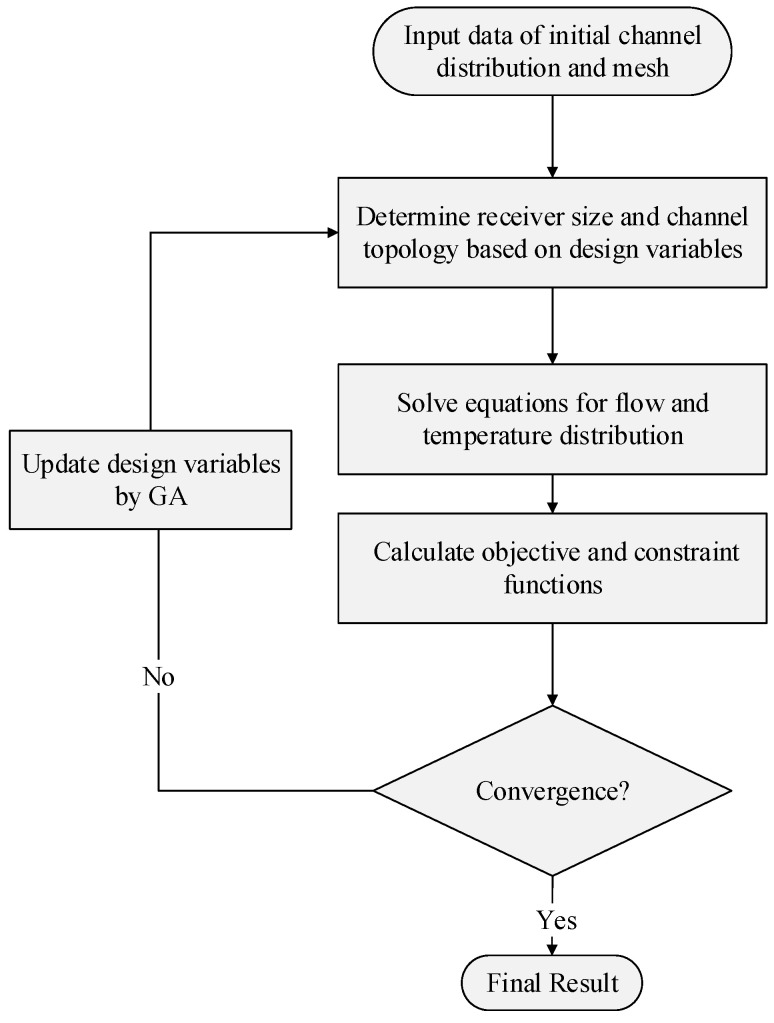
Flow chart of GA-based size and topology optimization of the thermal receiver.

The metaheuristic algorithm GA can search the entire design space without computing gradients, which makes it better at dealing with optimization problems with highly nonlinear objective functions and many local optimal solutions. However, a GA requires more computational cost than gradient-based optimization algorithms when dealing with optimization problems with many design variables. In this article, the optimal solution can be obtained at a lower time cost using a GA, based on the net-based thermal–fluid model with low computational cost. The GA is used in this work to adopt the optimizations for the fluid flow layout and receiver size as shown in Figure 5. In the follow-up examples, a population size of 200 is used in the GA, with a crossover fraction of 0.8 and a migration fraction of 0.2. The maximum number of generations is set to 100 times the number of design variables. The stopping criterion of the GA is when the average relative change in the best fitness function value is less than or equal to the tolerance.

The diameter and existence (topology) of channels are determined by the design variable ξ. In topology optimization, the diameter $d_{i}$ of the cross-section of the *i*-th branch is defined as:

(31) $$d_{i}\left(\xi \right)=\{\begin{array}{c} 0,\;\;\;\;\;\;\;\;\;\;\;\;\;\;\;\;\;\;\;\;\;\;\;\;\;\xi_{min}\leq \xi_{i}<\xi_{m}\\ d_{0}\cdot \xi_{i}+d_{1},\;\;\;\;\;\;\;\;\xi_{m}\leq \xi_{i}\leq \xi_{max} \end{array},$$

where $d_{0}$ and $d_{1}$ are the constant coefficients used to define the mapping relationship between the design variables and the diameter of channel branch, ξ is the topology design variable, $\xi_{m}$ is the threshold for deleting a branch, which is set to 0.5 here, and $\xi_{max}$ and $\xi_{min}$ represent the upper and lower limits of the design variables. If $\xi_{i}$ is less than $\xi_{m}$, the corresponding channel branch slightly contributes to the performance of this system and can be removed. If $\xi_{i}$ lies between $\xi_{m}$ and 1, this channel branch will be retained and its diameter will change as $\xi_{i}$ changes.

Then, the relationships between the receiver’s geometric size and the design variables are defined as follows:

(32) $$\{\begin{array}{c} d_{cav}=d_{2}\xi_{cav}+d_{3},\;\\ d_{ap}=d_{4}\xi_{ap}+d_{5},\\ \delta_{insu}=d_{6}\xi_{insu}+d_{7},\; \end{array}.$$

where $d_{2}$ to $d_{7}$ are the constant coefficients used to define the mapping relationship between the design variables ($\xi_{cav}$, $\xi_{ap}$ and $\xi_{insu}$) and receiver size.

Finally, the optimization problem can be described as follows:

$$\begin{array}{c} \mathrm{Find}\;\xi ={\left[\xi_{1}\;\xi_{2}\;\ldots \;\xi_{n}\;\xi_{cav}\;\xi_{ap}\;\xi_{insu}\right]}^{T},\\ \min J, \end{array}$$

(33) $$s.t.\;:\;\{\begin{array}{c} Kp=0,\\ \;AT=f\left(T\right),\\ \frac{\sum_{i=1}^{n}v_{i}}{V_{ini}}\leq V_{0},\\ \;\xi_{min}\leq \xi \leq \xi_{max}, \end{array}.$$

where *J* is the objective function, $\xi_{cav}\;$, $\xi_{ap}$, and $\xi_{insu}$ are the design variables determining $d_{cav}$, $d_{ap}$, and $\sigma_{insu}$; $v_{i}$ is the volume of each tube branch, $V_{ini}$ is the volume of the ground structure when the channel diameter takes the maximum value and $V_{0}$ is the volume constraint. $\xi_{max}$ and $\xi_{min}$ are set as 0 and 1, respectively, in the following optimization example.

Additionally, the MOO problem is considered to meet the balance between temperature non-uniformity, thermal efficiency, and pressure drop. The solution of this MOO problem includes a sequence of points of the Pareto frontier.

## 4. Results and Discussion

In this section, the relationship of thermal efficiency with temperature and receiver size are discussed to determine the range of optimization variables; moreover, a validation example for the proposed thermal–fluid model and an optimization example under non-uniform heat flux are given. It should be noted that the cavity shape, working fluid, and boundary condition settings of the examples in this section are all used as examples to demonstrate the effect of the optimization method and the thermal–fluid model developed in this paper, rather than being limited by these settings.

### 4.1. Analysis of Thermal Efficiency

According to the previous mathematical model of heat loss in Section 2.2, the relationship between thermal efficiency, temperature, and receiver size can be established for the subsequent optimization procedure.

#### 4.1.1. Relationship between Heat Loss and Cavity Temperature

**Figure 6:**
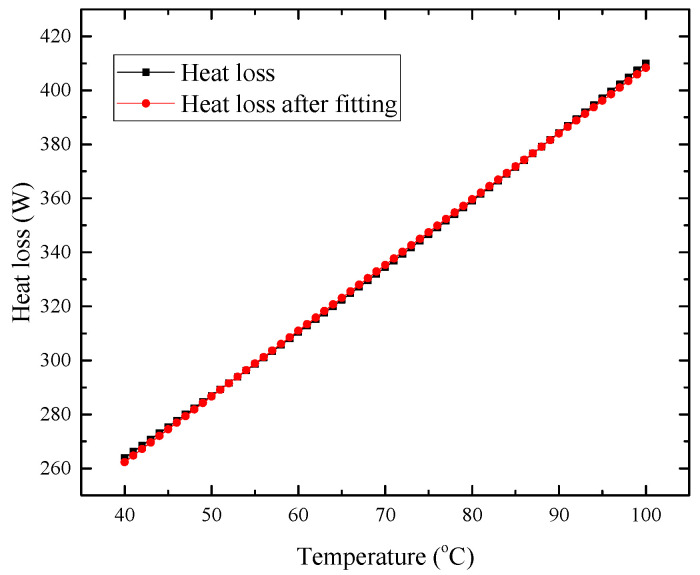
Result comparison between heat loss model and its fitting equation.

All the parameters, except temperature, are fixed to study the relationship between heat loss and cavity temperature. The important geometric parameters of the receiver are set as follows: $\sigma_{insu}=0.05\;m$, $d_{ap}=0.18\;m$, and $d_{cav}=0.30\;m$, $\theta =45^{o}$. The fitting temperature range is set at 40–100 °C. The other parameters with fixed values are shown in Table 2. As seen in Figure 6, using the heat loss model in Section 2.2, it can be found that thermal efficiency has an approximately linear relationship with receiver surface temperature. Therefore, a linear fitting method is used to obtain the relationship between temperature and thermal efficiency, thereby coupling the heat loss model with the thermal–fluid model. The equation after adopting the least square method of linear fitting is as follows:

(34) $$Q_{loss}=k_{loss}T+b_{loss},$$

(35) $$k_{loss}=\frac{\bar{TQ_{loss}}-\bar{T}\cdot \bar{Q_{loss}}}{\bar{T^{2}}-{\left(\bar{T}\right)}^{2}},$$

(36) $$b_{loss}=\bar{Q_{loss}}-k_{loss}\cdot \bar{T}.$$

In the above equations, $Q_{loss}$ is the heat loss, the unit is W, and *T* is the cavity temperature. Moreover, it should be noted that the geometric parameters $\sigma_{insu}=0.05\;m$, $d_{ap}=0.18\;m$, and $d_{cav}=0.30\;m$ set above are just an example corresponding to Figure 6. During the optimization process, the coefficient values $k_{loss}$ and $b_{loss}$ of the fitting equation will vary as the receiver geometry changes.

As shown in Figure 6, the maximum relative deviation of all 61 of these data points is 0.60% when comparing the results using the heat loss model in Section 2.2 with the results using the linear fitting equation, which proves that the fitting results are credible.

#### 4.1.2. Relationship between Thermal Efficiency and Receiver Size

In this section, the relationship between thermal efficiency and the geometric parameters of the receiver are discussed. The temperature is fixed at 50 °C to study the effect of receiver parameters on thermal efficiency.

**Figure 7:**
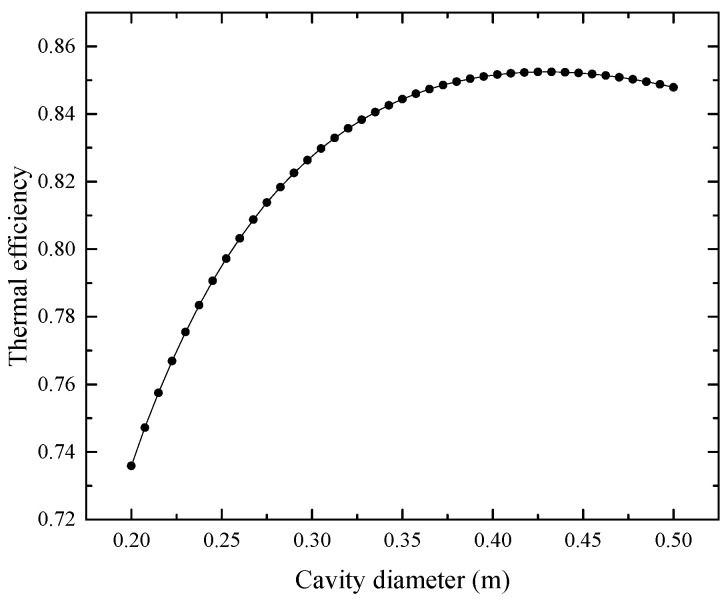
Influence of cavity diameter on thermal efficiency.

Figure 7 shows the impact of cavity diameter on thermal efficiency with cavity diameter varying from 0.20 m to 0.50 m. The insulation thickness and cavity aperture diameter are kept constant at 0.05 m and 0.18 m. As shown in Figure 7, the increase in the cavity diameter will make the thermal efficiency increase first and then decrease; therefore, there is a theoretical optimal value for cavity diameter.

**Figure 8:**
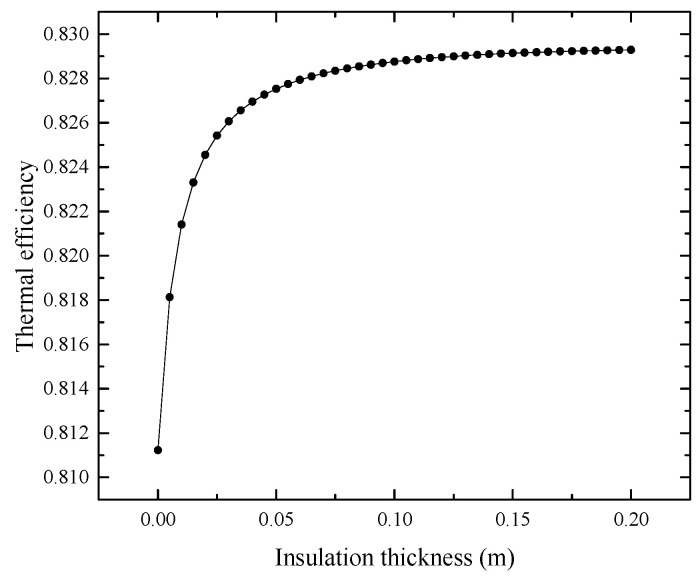
Influence of insulation thickness on thermal efficiency.

The influence of insulation thickness on thermal efficiency is portrayed in Figure 8 (insulation thickness changes from 0 m to 0.2 m). The cavity diameter and cavity aperture diameter remain at values 0.30 m and 0.18 m. It can be found that, within a suitable range, the larger the insulation thickness, the greater the thermal efficiency; however, the excessively large insulation thickness will reduce the thermal efficiency.

**Figure 9:**
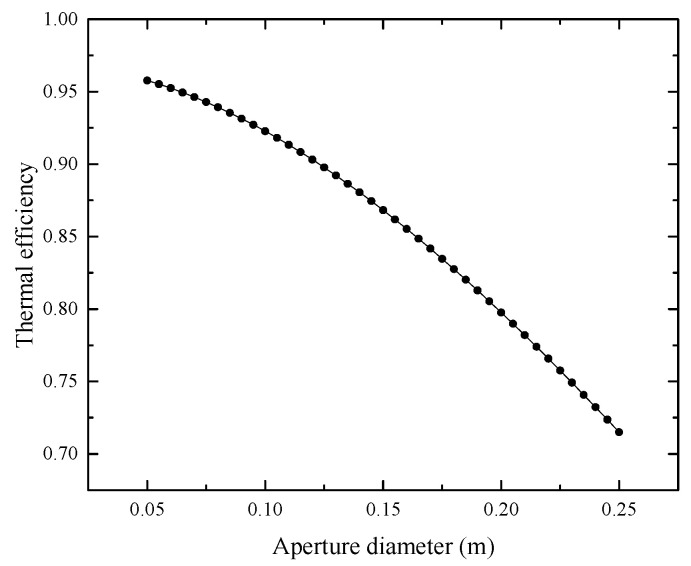
Influence of aperture diameter on thermal efficiency.

The thermal efficiency gradually decreases with increasing aperture diameter, as depicted in Figure 9 (aperture diameter changes from 0.05 m to 0.25 m). The insulation thickness and cavity diameter are kept at 0.05 m and 0.30 m.

The above relationship shows that there is optimization space in the receiver’s geometric size. According to the relationships of the three geometric parameters of the receiver, the ranges of the variation in receiver size in the optimization problem are determined: $d_{cav}\in \left[0.25,0.35\right]$, $\sigma_{insu}\in \left[0.025,0.075\right]$, and $d_{ap}\in \left[0.16,0.20\right]$. Therefore, the coefficients $d_{2}$ to $d_{7}$ in Equation (32) are defined as: 0.1, 0.25, 0.04, 0.16, 0.05, and 0.025, respectively.

### 4.2. Numerical Examples for Validation under Uniform Heat Flux

**Figure 10:**
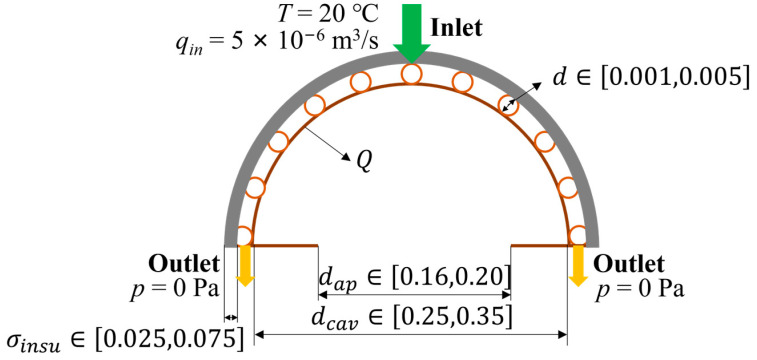
Design domain and boundary conditions.

In this section, a comparative numerical example is carried out, and the physical field results predicted by the net-based thermal–fluid model are compared with those predicted by the CFD software. All channel diameters are 2.5 mm (the diameters of the inlet and outlet are 5 mm). Water flows inside the channels surrounded by solid domains that consist of copper. The properties of water and copper are shown in Table 3. Furthermore, the design domain and boundary conditions are illustrated in Figure 10, where $d_{cav}=0.30\;m$, $\sigma_{insu}=0.05\;m$, and $d_{ap}=0.18\;m$. The heat flux is distributed across the interior surface of the cavity and represents the remaining heat of solar flux after subtracting all the heat losses. The CFD software employs the finite volume method in the numerical simulation of laminar flow for the Navier–Stokes equations under steady-state conditions and constant density. Additionally, heat conduction and convection are considered together, making it a coupled thermal–fluid problem. The segregated solver is employed within the software to solve this problem iteratively.

**Figure 11:**
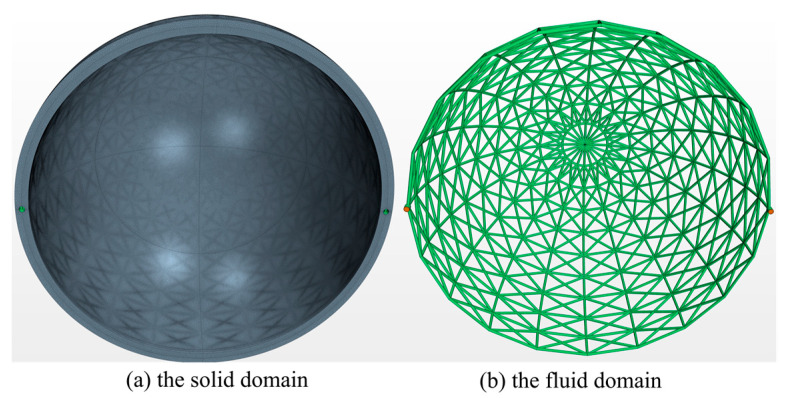
The mesh model for the numerical simulation.

**Figure 12:**
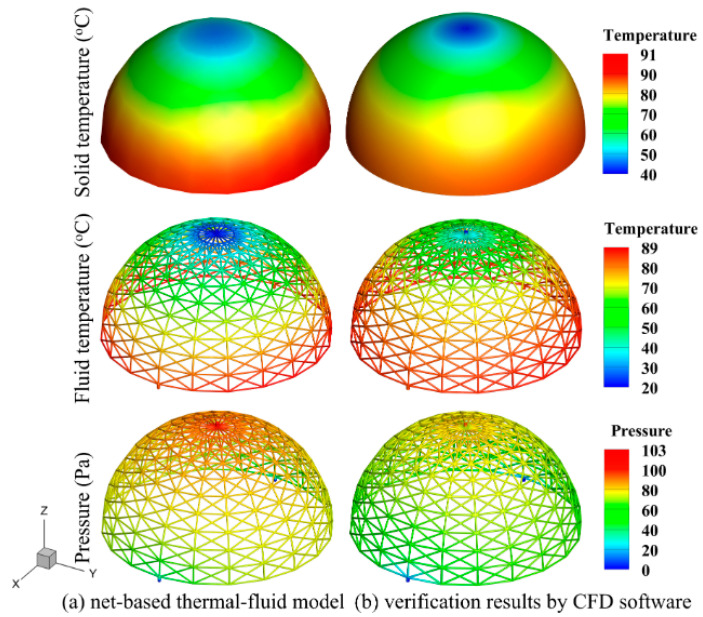
Comparisons between the temperature field (°C) and pressure field (Pa) predicted by net-based thermal–fluid model and those predicted by numerical simulation.

Grid-independence verification is conducted to ensure computational accuracy of the verification study by confirming that the number of meshes is sufficient. Specifically, 3 examples are computed using grid numbers of 3,464,861, 4,932,816, and 7,571,626, respectively. As shown in Table 4, comparing the results of the third example (which have the largest number of meshes) with those of the first and second groups, the distribution range of the absolute value of the relative deviation of the physical quantities concerned is found to be between 0.22% and 2.29%. Therefore, it can be concluded that the influence of the number of grids on the calculation results can be considered negligible when using 7,571,626 grids, and the required computational accuracy can be achieved. Figure 11 shows the mesh models of the solid and fluid domains, with a grid number of 7,571,626 for the three-dimensional simulation. Figure 12 presents comparisons between the physical fields predicted by the two methods. As shown, the distribution of temperature and pressure are in good agreement with the corresponding simulation results. The relative discrepancies between the 2 methods of the inlet pressure, average temperature, and maximum temperature of the solid domain are 9.62%, 4.83%, and 4.02%, respectively.

In addition, this embedded channel can effectively reduce the temperature difference between the interior and exterior solid surfaces of the cavity. The temperature of the two side surfaces is very close according to the data of the numerical simulation. The average temperature of the interior surface is 76.36 °C, and that of the exterior surface is 76.82 °C, with a relative difference of 0.59%. Therefore, it is feasible for both the temperature interior and the exterior cavity surface to be set to $T_{s-ave}$ in the assumption of the previous heat loss model in Section 2.2.

### 4.3. Optimization Results of the GA under Inhomogeneous Heat Flux

**Figure 13:**
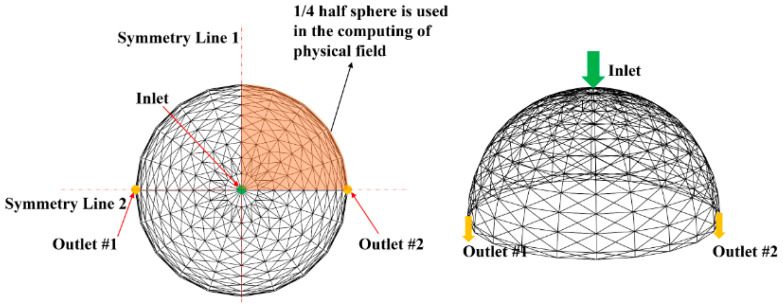
Schematic diagram of the symmetrical model.

**Figure 14:**
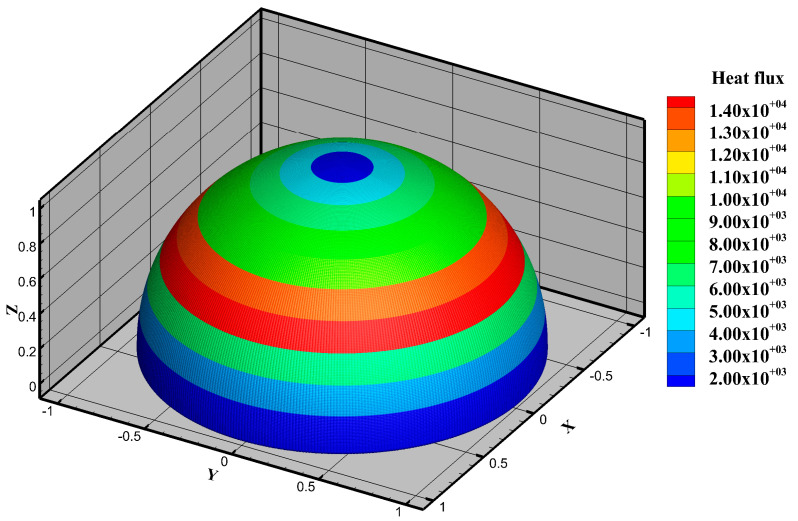
The distribution of inhomogeneous heat flux (W/m^2^) of the optimization result for *J*_2_.

In the optimization example, due to the symmetry of the entire design domain, a quarter of it is used as the design domain, as shown in Figure 13. Furthermore, inhomogeneous heat flux density is applied to the interior surface of the receiver and the distribution tendency of this heat flux is referenced from [43] but the specific values are different. Figure 14 illustrates the distribution of the inhomogeneous heat flux of *J*_2_ as an example. The heated surface is divided into 11 regions in these optimization examples. Although the heat flux distribution on the entire heating surface is nonuniform, the heat flux is assumed to be uniformly distributed in each sub-region, as shown in Equation (37):

(37) $$C\cdot q=Q_{rad-b}-Q_{loss},$$

where q is the distribution vector of heat flux, defined as: $q=\left[q_{1},q_{2},q_{3},\ldots ,q_{11}\right]$ and with a unit of W/m^2^. The coefficient vector of heat flux (W/m^2^) is assumed to be C=[1,1,2,4,9,8,6,5,4,3,1].

**Figure 15:**
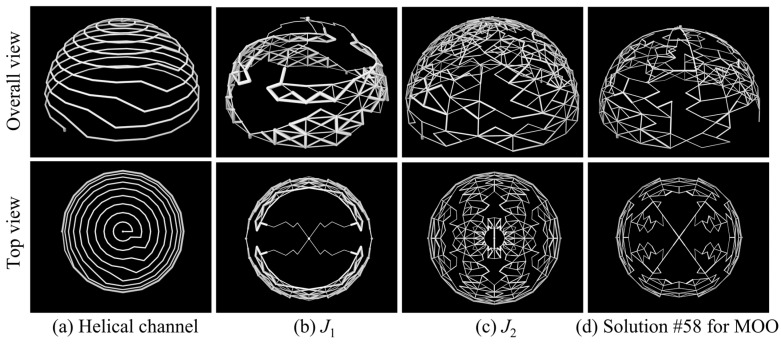
Topological structures of reference design (helical channel) and those of optimization results.

Using the optimization problem described in Section 3, the topological structures of the optimization results shown in Figure 15 are obtained, where $V_{0}$ is set to 0.10. A reference design (helical channel structure) with the same volume ratio as the optimized results is also implemented for comparison with the optimization results. The receiver’s geometric size for this reference design are as follows: $d_{cav}=0.30\;m$, $\sigma_{insu}=0.05\;m$, and $d_{ap}=0.18\;m$. It should be noted that the inlet and outlet channel diameters of all the optimization results and the reference design are fixed at 5 mm.

**Figure 16:**
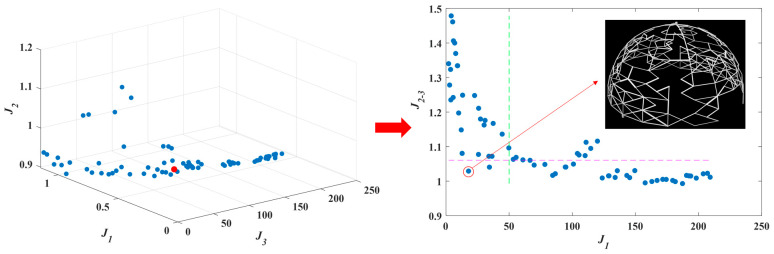
Pareto frontier for MOO problem and a suitable artificially selected solution.

Figure 16 shows the Pareto frontier for the MOO problem, where the dot in the red circle on the right (which corresponds to the red dot on the left) is the selected suitable solution, and is located at the 58th solution on the Pareto front (hereinafter referred to as solution #58). As shown in Figure 15d, solution #58 is a suitable artificially selected solution on the Pareto frontier of the MOO problem. The criteria for selecting the solution from the Pareto frontier in this article is determined as follows:

(38) $$\{\begin{array}{c} J_{3}=∆P/∆P_{0}\leq J_{3\_op}\\ J_{1-2}=\sqrt{J_{1}{}^{2}+J_{2}{}^{2}}\leq \sqrt{J_{1\_op}{}^{2}+J_{2\_op}{}^{2}} \end{array},$$

where $J_{1\_op}$~$J_{3\_op}$ are the optimization requirements of $J_{1}$~$J_{3}$, respectively, which are taken as 50, 0.75, and 0.75, respectively, in this example. For multi-objective optimization problems, every solution on the Pareto front is feasible, and there is no clear distinction between good and bad. In this paper, the optimization potential of each objective varies greatly. For example, the optimization improvement space of the $J_{2}$ objective is significantly smaller than that of the other objectives. Consequently, the priority of the $J_{2}$ objective is reduced, and the priority of the $J_{3}$ objective is increased when selecting the decision-making method for the final solution. Specifically, based on the formulated optimization requirements ($J_{1\_op}$~$J_{3\_op}$), the area in the solution set of the Pareto front that meets the requirements is selected, and the final solution is chosen from it. The final solution is used for visualization and compared with other single-objective optimization results. It should be noted that the decision-making method presented in this paper is just an example, and the degree of preference for different objectives may differ in actual use. Thus, an appropriate decision-making scheme can be tailored according to the different weights and priorities of the objective functions.

**Figure 17:**
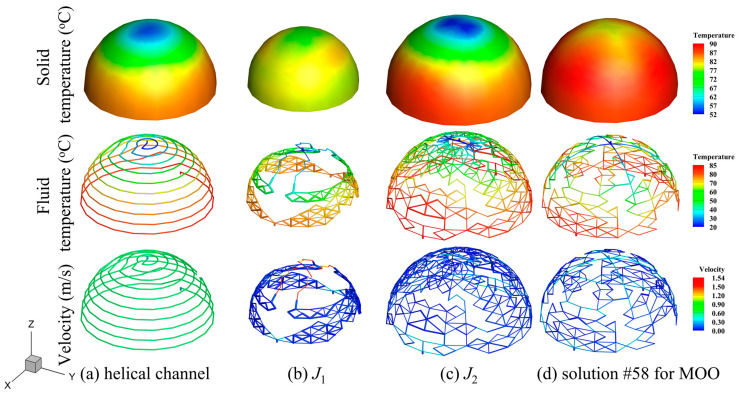
Comparisons between the physical fields of optimization results and those of the reference design (helical channel).

The comparisons between the physical fields of the optimization results and those of the reference design are demonstrated in Figure 17. It can be found that different optimization objectives lead to different topological features. Several indicators of the optimization results are shown in Table 5. The topology structure of the optimization results presents a multi-branch and multi-partition network-type channel feature, which is different from the conventional helical channel structure of the reference design. The optimization result of $J_{1}$ gradually bifurcates into an increasing number of sub-channels. Fluids with higher heat transfer capacity can be distributed into regions with larger temperature gradients, thereby reducing temperature inhomogeneity as much as possible. However, the receiver size of the $J_{1}$ result, shown in Table 5, is too small, resulting in low thermal efficiency. The optimization result of $J_{2}$ is to retain more branches with a larger receiver, but the average channel diameter is lower (see Table 5), thus increasing the region that can be reached by the flow and improving thermal efficiency under the constraint of volume ratio. The MOO result combines the characteristics of the above optimization results, and thus retains the advantages of low-temperature difference and high thermal efficiency with more suitable flow dissipation. Comparing the MOO result with the reference design result, the temperature standard deviation of the former decreases from 9.95 °C to 2.82 °C, a relative decrease of about 72%; the thermal efficiency increases from 79.10% to 79.25%, an increase of about 0.2%; and the pressure drop decreases from 14,980 Pa to 1832 Pa, a decrease of about 88%. However, in the optimization results shown in Figure 17, some areas form a structure with sharp corners instead of a streamlined structure, which is due to the ground structure of the net-based model in this paper. The model could be improved through follow-up research to solve the above drawback and give the channel shape a greater degree of freedom in optimization.

## 5. Conclusions

In this article, a thermal–fluid model based on a channel network was adopted to describe the flow and heat transfer processes of a thermal receiver with low computational cost, and to develop a topology optimization method using a GA to optimize the performance of the receiver. The thermal–fluid model was verified through comparison with the numerical simulation. Two single-objective topology optimizations and a multi-objective topology optimization were implemented for the cavity receiver and the channels in it under inhomogeneous heat flux. The following conclusions can be drawn.

(1) The physical field results of the net-based thermal–fluid model under uniform heat flux were compared with those of the numerical simulation to verify the accuracy of the model. The relative discrepancies of the inlet pressure, average temperature, and maximum temperature of the solid domain between the two were 9.62%, 4.83%, and 4.02%, respectively.

(2) The heat loss model was coupled with the thermal–fluid model using linear fitting to achieve topology optimization and size optimization of the receiver. In the comparative example, the linear fitting result had a maximum deviation of 0.6% compared with the calculated result of the heat loss model.

(3) Single-objective optimizations of temperature uniformity ($J_{1}$) and thermal efficiency ($J_{2}$) and a multi-objective optimization (MOO) that considered $J_{1}$, $J_{2}$ and the pressure drop were carried out using a GA. The optimization results provided better comprehensive performance than those of the reference design (helical channel) under the same conditions. Compared with the reference design, the temperature uniformity of the $J_{1}$ result was improved by 83%; the thermal efficiency of the $J_{2}$ result was improved by 3%; the MOO result illustrated a 72% and 0.2% improvement in temperature uniformity and thermal efficiency, respectively; and the pressure drop was reduced by 88%. Moreover, the topological characteristics of the optimization results were different from the conventional helical channel, and the optimization effect proved the effectiveness of the proposed topology optimization method.

## Figures and Tables

**Table 1:** The lower and upper bounds of geometric parameters in these optimization problems.

Design Variables	Definition	Lower Bound (mm)	Upper Bound (mm)
d	diameter of channels	1	5
$d_{cav}$	diameter of cavity	250	350
$d_{ap}$	diameter of aperture	160	200
$\delta_{insu}$	thickness of insulation	25	75

**Table 2:** Relevant parameters set in the examples.

Parameters	Definition	Values
$T_{\infty }$	Ambient temperature	20 °C
θ	Receiver inclination angle	45°
$\varepsilon_{s}$	Emissivity of receiver surface	0.83
$\alpha_{s}$	Absorptivity of radiative surface	0.75
$Q_{b}$	Solar beam radiation energy	300 W
$\tau_{i}$	Intercept factor	0.94
$\tau_{shad.}$	Shading factor	0.95
$\rho_{mirror}$	Mirror reflectivity	0.85
$V_{wind}$	Wind velocity	1 m/s
*F*	View factor	1

**Table 3:** Material properties for the fluid and solid.

Material	*ρ* [kg/m^3^]	*υ* [m^2^/s]	*k* [W/m K]	*c_p_* [J/(kg K)]
Water	997.56	8.91 × 10^−7^	0.62	4181.72
Copper	8940	-	386	386

**Table 4:** Grid independence verification.

Grid Number	$T_{s}$ [°C]	Deviation	$P_{in}$ [Pa]	Deviation
3,464,861	76.66	0.39%	93.21	−0.40%
4,932,816	76.58	0.29%	95.72	2.29%
7,571,626	76.36	-	93.58	-

**Table 5:** Comparison of indicators of optimization results.

Objective	Reference Case	*J*_1_	$J_{2}$	MOO
Inlet pressure (Pa)	14,980	15,093	1817	1832
Standard deviation of solid temperature (°C)	9.95	1.67	11.17	2.82
Thermal efficiency (%)	79.10%	72.26%	81.64%	79.25%
Total number of branches	202	359	667	405
Average channel diameter (mm)	3.23	2.56	1.90	1.68
$d_{cav}$(m)	0.30	0.25	0.33	0.31
$d_{ap}$(m)	0.18	0.20	0.16	0.17
$\delta_{insu}$(m)	0.05	0.03	0.07	0.05

## Data Availability

Not applicable.

## Nomenclature

**A**	Temperature matrix
$A_{s}$	Mean surface area (m^2^)
$A_{0}$	Area of receiver aperture (m^2^)
$c_{p}$	Specific heat (J kg^−1^ K^−1^)
**C**	Distribution vector of heat flux (W/m^2^)
d	Diameter of branch channel (m)
$d_{cav}$	Cavity diameter (m)
$d_{ap}$	Receiver aperture diameter (m)
$depth_{s}$	Cavity depth (m)
$D_{out}$	Exterior surface diameter of the receiver cavity (m)
$D_{ground}$	Diameter of the ground structure for the channel model (m)
$f_{t}$	Load vector for temperature field (W)
F	View factor
g	Gravitational acceleration (m s^−2^)
Gr	Grashhof number
$h_{out}$	Heat transfer coefficient for convection at the exterior of an insulated receiver (W m^−2^ K^−1^)
J	Objective function
$k_{\infty }$	Thermal conductivity of atmospheric air (W m^−1^ K^−1^)
$k_{insu}$	Thermal conductivity of insulation (W m^−1^ K^−1^)
K	Friction factor matrix
m	Node number
Nu	Nusselt number
$N_{e}$	Number of solid elements
$N_{RC}$	Radiation conduction number
Pr	Prandtl number
p	Pressure (Pa)
$p_{in}$	Inlet pressure (Pa)
$p_{0}$	Inlet pressure of the initial structure (Pa)
p	Pressure vector (Pa)
Q	Heat transfer capacity (W)
q	Volume flow (m^3^ s^−1^)
$q_{in}$	Inlet volume flow (m^3^ s^−1^)
r	Friction factor (Pa m^−6^ s^−2^)
Ra	Rayleigh number
Re	Reynolds number
$R_{th}$	Thermal resistance
$Std_{0}$	Standard deviation of the solid domain temperature for the initial structure (°C)
T	Temperature variable (°C)
T	Temperature vector (°C)
$T_{s}$	Interior cavity surface temperature (°C)
$T_{cav-surface}$	Exterior cavity surface temperature (°C)
$T_{s-ave}$	Average cavity surface temperature (°C)
$T_{\infty }$	Ambient temperature (°C)
$V_{0}$	Volume constraint
$V_{wind}$	Wind velocity (m s^−1^)
Greek symbols	
μ	Dynamic viscosity (Pa s)
ρ	Density (kg m^−3^)
ξ	Design variable in optimization problem
ξ	Vector of topology design variables
$\xi_{m}$	Threshold for deleting a branch
θ	Receiver inclination angle (°)
ε	Emissivity of receiver surface
α	Absorptivity of receiver surface
β	Volume expansion coefficient (K^−1^)
$\sigma_{insu}$	Thickness of insulation (m)
$\tau_{i}$	Intercept factor
$\tau_{shad}$	Shading factor
$\rho_{mirror}$	Mirror reflectivity
$\eta_{th}$	Thermal efficiency
$\eta_{0}$	Thermal efficiency of the initial structure
Subscripts	
amb	Ambient
avg	Average
ap	Aperture
cav	Cavity
cond	Conduction heat transfer
conv	Convection heat transfer
eff	Effective
em	Emission
forced	Forced condition
insu	Insulation
ini	Initial
mirror	Mirror reflective surface
max	Maximum
min	Minimum
nat	Natural condition
out	Outer surface
rad	Radiative heat transfer
ref	Reflection
